# Anemia, Thrombocytopenia, and Changes in Biochemical Parameters Occurring in Patients with Uncomplicated *Plasmodium falciparum* Malaria: Data Analysis from Antimalarial Efficacy-Randomized Trials in Dakar and Kaolack Regions, Senegal

**DOI:** 10.1155/2022/1635791

**Published:** 2022-12-22

**Authors:** Khadime Sylla, Roger Tine, Doudou Sow, Souleye Lelo, Annie Abiola, Jean Louis NDiaye, Magatte NDiaye, Kuaku Folly, Léon Amath NDiaye, Oumar Gaye, Babacar Faye

**Affiliations:** Département de Parasitologie-Mycologie, Faculté de Médecine, Université Cheikh Anta DIOP de Dakar, Senegal

## Abstract

**Background:**

Artemisinin-based Combination Therapies (ACTs) are widely used in the treatment of uncomplicated malaria. *Plasmodium falciparum* infection is often accompanied by disturbances of hematological and biochemical parameters. The objective of this study was to evaluate the changes in biochemical and hematological parameters during uncomplicated malaria in patients treated with ACTs.

**Methods:**

Data from patient with uncomplicated *Plasmodium falciparum* malaria were pooled from different open-randomized trial evaluating the efficacy of Artesunate-Mefloquine (ASMQ), Artesunate-Amodiaquine (ASAQ), Artemether-Lumefantrine (AL), and Dihydro-artemisinin-Piperaquine (DHAPQ) combinations. Biochemical (transaminases, creatinine, and bilirubin) and hematological (hemoglobin and platelet levels) parameters were performed at baseline (D0) and at day 7 after treatment (D7). Data were analyzed as both continuous and categorical variables with 95% confidence interval. Risks and trends were calculated using multivariate logistic random effect models.

**Results:**

A total of 720 patients with completed biological data were included in the analysis (320 in the AL arm, 160 in the ASMQ arm, 120 in the DHAPQ arm, and 88 in the ASAQ arm). The mean age of the patients was 9.43 ± 9.1 years. Male subjects represented 58.47% (sex ratio was 1.4 for males). The mean hemoglobin level at inclusion (D0) was 9.79 g/dl and anemia (Hb < 11 g/dl) was 71.43% (aOR = 1.16 [0.68 − 1.98]*p* = 0.57). At D7, hemoglobin level was 9.63 g/dl and anemia was significantly more frequent (78.29% [*p* = 0.002]). The mean platelet count at day 0 was 154075.5 platelets/mm^3^ of blood and 339328.7 platelets/mm^3^ at day 7. Thrombocytopenia was about 53.61% and was associated with malaria (aOR = 3.4 [2.18 − 5.3]*p* < 10^−3^). 19.58% of patients had abnormal ALT and 40.28% had abnormal AST at D0. 27.22% of patients had normal bilirubin at D0. Renal function was normal in all patients in the study. Normalization of transaminases was noted between D0 and D7. The percentage of subjects with normal bilirubin increased between D0 and D7. Renal function did not vary significantly between D0 and D7.

**Conclusion:**

Results from this analysis showed that subjects with high parasitaemia had a greater risk of anemia and thrombocytopenia. Artemisinin combinations were well-tolerated as no major biological disturbances were noted. The effects of ACTs on hematologic and biochemical parameters were not different.

## 1. Introduction

Malaria is a major public health problem in developing countries. According to World Health Organization (WHO), 241 million cases of malaria are recorded each year and there are approximately 627000 deaths. More than 95% of these cases and 96% of the deaths occur in Africa and mainly in children under 5 years old (80% of all malaria deaths in the region [1].

Apart from this very high morbidity and mortality, malaria has many consequences on the health of populations. Among these consequences are disturbances of hematological and biochemical parameters, but these disturbances are not well studied.

Several modifications of hematological parameters have been reported by Laura et al. when evaluating clinical and hematological indices during malaria [2].

It has been shown that the most frequent hematological changes during malaria are anemia, thrombocytopenia, and leukopenia. Other studies have also shown the influence of malaria on hematological parameters and the causal link between anemia and thrombocytopenia [3–5].

Malaria often leads to disturbances in liver function. It has been shown that during *Plasmodium falciparum* malaria, there is a release of hydroxide and peroxide radicals. This release of radicals would be at the origin of a disturbance of hepatic function resulting in an elevation of transaminases [6].

In addition to changes in hematological parameters and disturbances in hepatic function, malaria is often the cause of dysfunction in renal function manifested by acute tubular necrosis with an increase in creatinine clearance [7].

To fight malaria and its consequences, the WHO has recommended since 2001 the cessation of monotherapy and the use of Artemisinin-based Combination Therapies (ACTs) [8].

Following WHO recommendations, Senegal, through its National Malaria Control Program (NMCP), changed its policy for the management of uncomplicated malaria cases by adopting artemisinin-based combination therapy in 2006 [9].

Several studies have shown that artemisinin-based combination therapies are effective and well-tolerated in the treatment of uncomplicated malaria.

As a line extension of the ongoing surveillance system and to better document the modifications of biological parameters during malaria infection, this study was assessing the effect of *Plasmodium falciparum* malaria on certain biological markers in the context of efficacy clinical trials on artemisinin combination therapy in Senegal.

## 2. Materials and Methods

### 2.1. Study Design and Period

This study was designed within the framework of open-randomized trials evaluating the efficacy and safety of artemisinin-based combination therapies in Senegal. The combinations Artesunate-Mefloquine (ASMQ), Artesunate-Amodiaquine (ASAQ), Artemether-Lumefantrine (AL), and Dihydro-artemisinin-Piperaquine (DHAPQ) were studied during randomized open trials conducted in the health districts of Dakar (Deggo health post) and Kaolack (Medina Baye and Keur Socé health post). In Dakar, the study was conducted in Deggo health post which is located at 20 km from the capital city. In Dakar, malaria incidence is between 5% to 15% habitants. Kaolack region is located in the central part of Senegal, 200 km from Dakar. The study was carried out in Keur Socé and Medina Baye health posts where malaria incidence is more than to 15% habitants.

In these areas, malaria is highly seasonal during the rainy season (July to October) with a peak of transmission from September to December. *P. falciparum* is the predominant species and transmission is mainly due to *Anopheles gambiae s.l.*

### 2.2. Study Population

Patients consulting at the outpatient offices of different health posts were included in these open-randomized trail studies. Patients were eligible to be enrolled after giving an informed consent, patients with age above 6 months who attended the health post with a history of fever in the preceding 24 hours or confirmed fever (axillary temperature ≥ 37.5°C), with uncomplicated *P. falciparum* malaria, parasitaemia between 1000 and 100000 trophozoites/*μ*l, and patients able to take oral medication. Patients with severe and complicated malaria, according to the WHO definition [10], or monoinfection by another species or mixed infestation, severe vomiting, severe malnutrition, a woman with positive pregnancy test, patients who had a history of allergy to study drugs, or did not give informed consent were excluded from the study.

### 2.3. Study Description

A 28-day follow-up was performed for every patient included in the study, using the WHO 2003 protocol for assessing the efficacy of antimalarial drugs. Thick smear and thin smear were performed in all study patients at baseline and at the other follow-up days. Hematological and biochemical parameters were measured at day 0 (D0) and day 7 (D7) of follow-up. Blood samples were taken by venipuncture. For each patient, 5 ml of blood was taken in an EDTA tube (Ethylene Diamine Tetra Acetic) for the determination of the hematological parameters using Hematology Analyzer ABX Micros ES 60, Horiba. The same amount of blood was collected in a dry tube to study biochemical parameters with A15 Chemistry Analyzer, BioSystems.

The treatments administered during the study were artemether-lumefantrine combination (120 mg artemether plus 20 mg lumefantrine in 2 doses per day), artesunate-mefloquine (50 mg artesunate and 125 mg mefloquine in one sachet per day), dihydro-artemisinin-piperaquine (320 mg DHA plus 40 mg PQ as a single dose per day), and artesunate-amodiaquine (4 mg/kg/day artesunate plus 10 mg/kg/day amodiaquine as a single dose per day). All combinations were administered for 3 days. Drug-event relationship could not be attributed as it has not been assessed systematically across the clinical trials. In this analysis, we just described the risk factors associated with anemia and thrombocytopenia and the evolution of biochemical parameters from day 0 to day 7 in different treatment groups.

### 2.4. Data Collection

Sociodemographic and biological data were extracted from the different databases from clinical open-randomized trials performed in Senegal. The variables studied were age defined in two categories (under 15 years and over 15 years); sex (male or female); treatment (ASAQ, AL, DHAPQ, and ASMQ); parasitaemia classified as low (5000 trophozoites/mm^3^), moderate (5000-25000 trophozoites/mm^3^), and high (>25000 trophozoites/mm^3^); hemoglobin level in g/l; and platelet count/mm^3^ of blood and biochemical markers such as transaminases (ALT and AST in IU/l), bilirubin (mg/dl), and creatinine (mg/l).

### 2.5. Data Entry and Analysis

Data were entered into Excel and analyzed using Stata MP 16 software. Quantitative variables were described in terms of mean and standard deviation. For qualitative variables, a description in terms of number of participants and percentage of data completed was performed. Data were analyzed by estimation of difference in proportion according to a 95% confidence interval. Groups were compared using chi-square test or Fisher exact test for categorical variables and Student's *t* test for continuous variables when these tests were applicable. Otherwise, nonparametric tests (Mann–Whitney and Kruskall-Wallis) were used.

A multivariate analysis was performed to study the factors that could be associated with thrombocytopenia and anemia at inclusion. The study of the variation of hematological and biochemical parameters between D0 and D7 was done by pairwise analysis using the Bonferroni test. Statistical significance for all tests was set at 0.05 (*p* value <0.05 two side).

### 2.6. Ethical Considerations

All studies were conducted according to the Declaration of Helsinki and existing national legal and regulatory requirements. All protocol for randomized trial were approved by the Senegalese National Ethical Committee (Conseil National d'Ethique et de Recherche en Santé) [11–13].

## 3. Results

### 3.1. Characteristics of the Patients at Inclusion

Overall, 720 patients with uncomplicated *P. falciparum* malaria infection were randomized to receive either AL (*n* = 352) or ASAQ (*n* = 88), DHAPQ (120), and ASMQ (160). The age of the patients ranged from 1 year to 65 years with a median of 9.43 years. According to age category, study population was mainly represented by children under 15 years of age with a total number of 604 (83.9%). The adult subjects (age > 15 years) represented 116 patients (16.1%). The sex ratio was 1 for males (Table 1).

### 3.2. Biological Profile of Patients at Inclusion

Parasitaemia ranged from 1020 to 105000 trophozoites/*μ*l blood with a median parasitaemia of 27250 trophozoites/*μ*l. Depending on the treatment group, parasitaemia ranged from 1020 to 98845 trophozoites/*μ*l blood with a median of 22400 trophozoites/*μ*l blood in the ASMQ group. In the AL group, parasitaemia ranged from 1025 to 100000 trophozoites/blood unit with a median of 27509 trophozoites/blood unit. In the ASAQ group, parasitaemia ranged from 1040 to 988000 trophozoites/*μ*l blood with a median of 19046 trophozoites/*μ*l. In the DHAPQ arm, parasitaemia ranged from 1100 to 98291 trophozoites/*μ*l blood with a median of 32373 trophozoites/*μ*l blood.

The mean hemoglobin level at inclusion was 9.79 g/dl and the prevalence of anemia with a hemoglobin level below 11 g/dl was 71.43%. Stratifying on treatment, the mean hemoglobin level at inclusion was 10.03 g/dl in DHAPQ and AL groups versus 9.63 g/dl and 8.57 g/dl in the ASMQ and ASAQ groups. Anemia (hemoglobin level less than 11 g/dl) was more frequent in the ASAQ (86.59%) and ASMQ (76.51%) groups. In the AL and DHAPQ groups, the prevalence of anemia was 66.96% and 67.24%, respectively.

Overall, the mean platelet count was 154075.5 platelets/mm^3^ of blood. Depending on the treatment group, the platelet count at inclusion was 195936 platelets/mm^3^ blood and 160870 platelets/mm^3^, respectively, in the ASAQ and AL groups. This number was lower in the DHAPQ and ASMQ groups with 126493 platelets/mm^3^ and 136788 platelets/mm^3^ of blood, respectively. The incidence of thrombocytopenia (platelet count of less than 150000/mm^3^ of blood) was 53.61% at inclusion. Thrombocytopenia was estimated at 63.33%, 56.25%, 50%, and 36.36% in the DHAPQ, AL, ASMQ, and ASAQ groups, respectively.

Liver function was normal in most of the subjects included in this analysis. Only 19.58% of patients had abnormal ALT and 40.28% had abnormal AST. A mean ALT level of 31.6 IU/l was found in patients in ASMQ group. In DHAPQ, AL, and ASAQ groups, the mean ALT was 27.3 IU/l, 28.1 IU/l, and 29.1 IU/l, respectively. A normal ALT level was found in more than 80% of patients in DHAPQ, AL, and ASAQ groups. This level was lower (70.63%) in the ASMQ group. A mean AST level of 33.05 IU/l was found in DHAPQ group compared to AL, ASAQ, and ASMQ groups where the mean AST level was 42.3 IU/l, 42.8 IU/l, and 50.74 IU/l, respectively. The AST level was normal in 80.83% of patients in DHAPQ group, 61.65%% in AL group, 42% in ASMQ group, and 54.55% in ASAQ group. At inclusion, the mean bilirubin level was 1.01 mg/dl in DHAPQ and AL groups, 0.85 mg/dl in ASAQ group, and 0.9 mg/dl in ASMQ group. Only 27.22% of patients had normal bilirubin levels.

In general, all subjects included in the study had normal renal function. Mean creatinine levels were 6.1 mg/l, 5.8 g/l, 7.7 mg/l, and 4.8 mg/l in the ASMQ, ASAQ, DHAPQ, and AL groups, respectively. Results are described in Table 2.

### 3.3. Factors Associated with Anemia and Thrombocytopenia at Inclusion (Day 0)

The results from the analysis showed that there was no association between age and anemia and between gender and anemia. However, an association between parasitaemia and anemia was found. Subjects with high parasitaemia had a greater risk of anemia (aOR = 1.16 [0.68 − 1.98]*p* = 0.57) than subjects with low and moderate parasitaemia. An association between age and thrombocytopenia was noted in this study. Thus, patients older than 15 years had twice the risk of developing thrombocytopenia (aOR = 2.32 [1.5 − 3.6]*p* = 0.0002) compared to subjects younger than 15 years. Results from the analysis also showed an association between gender and thrombocytopenia. Male subjects had a 10% greater risk of developing thrombocytopenia (aOR = 1.1 [0.81 − 1.5]*p* = 0.52) compared to female subjects. An association between parasitaemia and thrombocytopenia was also found. Subjects with moderate parasitaemia had twice the risk of thrombocytopenia (aOR = 2.01 [1.26 − 3.2]*p* = 0.0035) compared to subjects with low parasitaemia. Subjects with high parasitaemia had a 3-fold increased risk of thrombocytopenia (aOR = 3.4 [2.18 − 5.3]*p* < 10^−3^) compared to subjects with moderate and low parasitaemia (Table 3).

### 3.4. Variation in Hematological and Biochemical Parameters between Day 0 and Day 7

**Figure 1:**
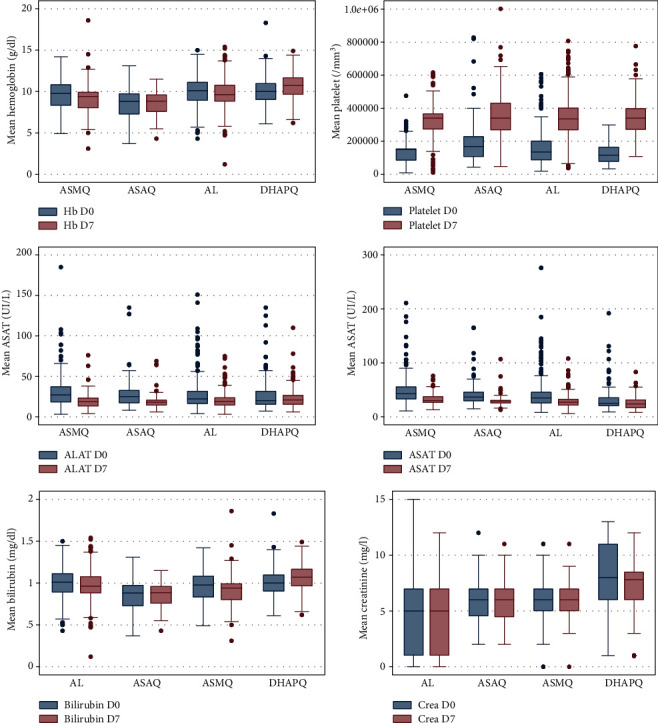
Mean hematologic and biochemical values by treatment at D0 and D7 in patients with uncomplicated *Plasmodium falciparum* malaria. Units for hemoglobin (g/dl), units for platelets (/mm^3^), units for ALAT and ASAT (IU/l), units for bilirubin (mg/dl), and units for creatinine (mg/l).

A decrease in hemoglobin level was observed between D0 and D7 from 9.76 g/dl to 9.63 g/dl. The difference in mean between D0 and D7 was 0.14 g/dl (*p* = 0.03). Depending on the treatment group, hemoglobin level decreased from D0 to D7 in AL and ASMQ groups. In contrast, in DHAPQ and ASAQ groups, an increase was noted. In DHAPQ group, the mean hemoglobin level increased from 10.03 g/dl to 10.7 g/dl between D0 and D7 (*p* = 0.001). In ASAQ arm, mean hemoglobin level was lower at D0 (8.57 g/dl) compared to D7 (8.63 g/dl) without significant difference (*p* = 0.76). In AL group, mean hemoglobin level was 10.03 g/dl at D0 versus 9.7 g/dl at D7 (*p* = 0.049). A decrease in hemoglobin level was noted between D0 and D7 in the ASMQ group from 9.63 g/dl to 9.1 g/dl (*p* = 0.0001) (Figure 1).

Pairwise analysis showed an increase in hemoglobin from D0 to D7 in DHAPQ and ASAQ groups. The mean difference mean was −0.062 ± 0.2 g/dl [95% CI (-0.47-0.35)] and −0.65 ± 0.14 g/dl [95% CI (-0.94--0.37)], respectively, in ASAQ and DHAPQ group (*p* = 0.07). In contrast, in AL and ASMQ groups, a decrease of 0.27 ± 0.08 g/dl [95% CI (0.1-0.44)] and 0.54 ± 0.08 g/dl [95% CI (0.27-0.81)] was noted in AL and ASMQ groups, respectively (*p* = 0.56). Between AL (0.27 g/dl) and DHAPQ (-0.062 g/dl) groups and DHAPQ and ASMQ groups (0.54 g/dl), the differences were significative (*p* < 10^−3^) (Table 4).

**Figure 2:**
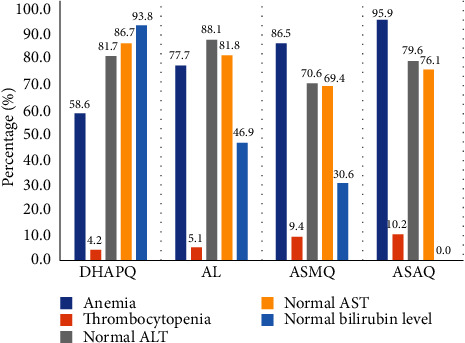
Prevalence of anemia, thrombocytopenia, normal liver enzyme, and bilirubin at day 7 after malaria treatment.

The prevalence of anemia increased from 71.43% to 78.29% between D0 and D7 (*p* value = 0.002). Anemia at D7 in ASAQ and ASMQ groups was 95.9% and 86.5%. In DHAPQ and AL groups, it was 58.6% and 77.7% (Figure 2).

Figure 1 shows significant increase of platelet count from D0 to D7 in all treatment groups (*p* < 10^−3^). At baseline, more than 50% of patients in each group had thrombocytopenia. However, at D7, a significant decrease in thrombocytopenia was observed in different treatment groups. At D7, thrombocytopenia was more frequent in ASAQ and ASMQ groups with 10.2% and 9.4%, respectively (Figure 2).

Pairwise analysis showed an increase in platelet count between D0 and D7 with significant differences comparing AL-DHAPQ and DHAPQ-ASMQ groups (Table 4).

Normalization of transaminases was observed between D0 and D7 in all treatment groups. The percentage of subjects with a normal ALT level was higher at D7 after treatment (86.5%) compared to D0 (80.4%). Patients with normal ALT were higher in AL group (88.1%) and DHAPQ group (81.7%). The number of patients with normal AST increased significantly between D0 and D7 in all treatment groups (Figure 2).

No significant difference was observed between ASMQ (11.7 IU/l) and AL (7.6 IU/l) (*p* value = 1), between ASAQ (9.3 IU/l) and AL (*p* = 1) and between ASAQ and ASMQ (*p* value = 1). ALT levels decreased by 3.15 IU/l in the DHAPQ group. When comparing DHAPQ to the other groups, the difference was significant (*p* < 0.05). The mean AST decreased by 12.8 IU/l in ASAQ group versus 14.4 IU/l in AL group (*p* value = 0.79). The same trends were observed between ASAQ and ASMQ groups (*p* = 0.29), between ASAQ and DHAPQ (*p* = 0.07), and between AL and DHAPQ (*p* = 0.6). The differences were significative between ASMQ and AL and between ASMQ and DHAPQ (*p* < 10^−3^) (Table 5).

Mean bilirubin decreased significantly between D0 and D7 in all treatment groups except DHAPQ group where an increase was noted but not significative (Figure 1).

The percentage of subjects with normal bilirubin increased between D0 and D7 in patients in the DHAPQ, AL, and ASMQ groups. In contrast, in the ASAQ group, any subject had normal bilirubin levels between D0 and D7 (Figure 2).

Renal function did not vary significantly between D0 and D7 in the 4 treatment groups. Between D0 and D7, mean creatinine production decreased by 0.35 mg/l in the ASAQ group (*p* = 0.0001). In the AL, DHAPQ, and ASMQ groups, creatinine levels decreased by 0.31 mg/l, 0.89 mg/l, and 0.41 mg/l, respectively, between D0 and D7 (*p* < 10^−3^) (Figure 1).

Pairwise analysis between the different groups showed significant differences (*p* < 10^−3^).

Concerning liver function, normalization was noted between D0 and D7 (Table 5).

## 4. Discussion

In sub-Saharan Africa, malaria is still a major public health problem despite all the efforts and means deployed to fight this disease. Morbidity and mortality related to malaria are very important and many consequences on the health of population, such as anemia, thrombocytopenia, and disturbances of the renal and hepatic functions are observed.

To address this situation, WHO has recommended the use of Artemisinin-based Combination Therapies (ACTs) for the management of uncomplicated malaria. These ACTs have proven to be effective and well tolerated. In Senegal, ACTs have been scaled up since 2006. The scale-up of ACTs must be accompanied by the establishment of a pharmacovigilance system to better document possible side effects of ACTs. This study was conducted to evaluate the changes in biochemical and hematological parameters during malaria infection in patients treated with ACTs.

The results from this analysis have demonstrated an association between parasitaemia and anemia. Subjects with moderate parasitaemia had a 10% greater risk of anemia (aOR = 1.16 [0.68 − 1.98]*p* = 0.57). The more the parasitaemia is high, the more the hemoglobin level is low.

Similar results have been shown by other authors. In Senegal, Tine et al. when studying the associations between malaria, erythrocyte polymorphism, malnutrition, and anemia in children under 10 years of age showed that malaria was associated with anemia (aOR = 5.23; 95% CI [1.1-28.48]) [14].

In Burkina Faso, Ouedraogo et al. when assessing the prevalence of malaria and anemia showed that anemia was significantly associated with malaria (*p* < 0.001) [15]. Other studies conducted in Ghana by Ehrhardt et al. and Agyei et al. have found an association between malaria and anemia (OR = 1.68 [1.38 − 2.04]) and (OR = 1.6 [1.08 − 2.35]), respectively [16, 17].

Same tendencies were noted in Democratic Republic of Congo and Ethiopia where Hedberg et al. and Deribew et al. have demonstrated a significative association between malaria and anemia in children under 5 years old [18, 19].

The results of our study are in line with what was previously described in Kenya where Maina et al. and Akhwale et al. have noted that anemia was significantly associated with malaria [20, 21].

An association between parasitaemia and thrombocytopenia was noted in our study. Subjects with moderate parasitaemia had a 2-fold increased risk of thrombocytopenia (aOR = 2.01 [1.26 − 3.2]*p* = 0.0035). Those with high parasitaemia had a 3-fold increased risk of thrombocytopenia (aOR = 3.4 [2.18 − 5.3]*p* < 10 − 3) compared to those with moderate and low parasitaemia. Same trends concerning the association between malaria and thrombocytopenia were previously described by in sub-Saharan Africa [22, 23]. Thrombocytopenia was identified as an independent predictor of death (OR = 13.3, 95% CI: 3.2–55.1) [22].

Zwang et al., when comparing the changes in hematologic parameters occurring in patients with uncomplicated *P. falciparum* malaria in sub-Saharan Africa, have noted that the risk of thrombocytopenia was generally high at inclusion and decrease after treatment with antimalarial drugs (during follow up) [24].

Concerning the liver enzymes, a normalization of transaminases was observed between D0 and D7 in all treatment groups The number of patients with normal transaminase was higher at day 7 (86.4% for ALT and 79.2% for AST) compared to the enrollment (28% for ALT and 59.7% for AST). This has been previously described by Sylla et al. in Senegal [11].

An elevation of liver enzymes was noted in Nigeria in patients with uncomplicated *Plasmodium falciparum* malaria [25]. This can be explained by the fact that malaria caused liver dysfunction resulting in the release of these enzymes into the blood. Other studies have demonstrated similar results [26, 27].

Regarding the mean of creatinine, no significant variation was observed between day 0 and day 7 in the different treatment arms. However, a significant decrease of bilirubin level was noted at day 7 in the three treatment arms. These results are in line with what were previously noted in Senegal [11–13].

## 5. Conclusion

Results from this analysis confirm the complexity of explaining hematological and biochemical changes occurring in acute malaria. The results noted patients with high parasitaemia had a greater risk of anemia and thrombocytopenia. No significant difference was noted between the different treatment groups at day 7 in term of ALAT and ASAT level as well as bilirubin and creatinine concentration. However, this analysis cannot distinguish between the changes occurring while patients recover from uncomplicated malaria and those from drug adverse reactions. Analyzing these results is not simple and will require confirmation on extended databases. For this reason, it will be necessary to obtain and collect information from as many studies. Finally, establishing reference laboratory ranges that are locally relevant should be a priority.

## Figures and Tables

**Table 1:** Baseline characteristics of study participant at the inclusion (*n* = 720).

Variables	Frequency (n)	Percentage (%)	95% CI
*Age group*			
<15 years	604	83.89	80.95-86.46
≥15 years	116	16.11	13.54-19.05
*Gender*			
Male	421	58.47	54.77-62.09
Female	299	41.53	37.91-45.23
*Treatment group*			
ASMQ	160	22.22	19.17-25.47
AL	352	48.89	45.18-52.61
DHAPQ	120	16.67	14.06-19.64
ASAQ	88	12.22	9.96-14.89

**Table 2:** Biological characteristics of patients at inclusion.

Variable	Global distribution	DHAPQ	AL	ASMQ	ASAQ
Parasitaemia (median ± trophozoites/*μ*l)	27250	32373	27509	22400	19046
Hemoglobin (mean g/dl)	9.76 ± 1.9	10.03 ± 1.8	10.1 ± 1.9	9.63 ± 1.7	8.57 ± 2.1
Anemia (Hb < 11 g/dl) (%)	71.43	67.24	66.96	76.51	86.59
Platelets (/mm^3^)	154075	126493	160870.7	136788	195936
Thrombocytopenia (<150000/mm^3^)	53.61	63.33	56.25	50	36.36
ALT (IU/l, mean ± SD)	28	27.3 ± 21	28.1 ± 20	31.6 ± 22	29.1 ± 22
Patients with ALT < 40 (%)	80.42	85	83.24	70.63	80.68
AST (IU/l, mean ± SD)	42	33.05 ± 25.8	42.3 ± 32	50.74 ± 30	42.8 ± 26
Patients with AST < 40 (%)	59.72	80.83	61.65	42.5	54.55
Mean bilirubin (mg/dl)	0.97	1.01 ± 02	1, 01	0.9 ± 0.2	0.85 ± 0.2
Patients with normal level of bilirubin	27.22	55.83	25.57	24.38	00
Mean creatinine (mg/l)	5.6 ± 3	7.7 ± 3.6	4.8 ± 3.1	6.1 ± 1.8	5.8 ± 1.8

**Table 3:** Associated factors with anemia and thrombocytopenia at inclusion.

	Number (%)	OR^∗^ (95% CI)	ORa^∗^ (95% CI)	*p* value
Anemia (Hb < 11 g/dl) *n* = 490				
*Age group*				
<15 years	437 (72.35%)	1	1	
≥15 years	53 (45.67%)	0.26 (0.17–0.39)	0.25 (0.16-0.38)	<10^−3^
*Gender*				
Male	208 (42.45%)	1	1	
Female	282 (69.5%)	0.84 (0.59-1.18)	0.77 (0.54-1.1)	0.16
*Parasitaemia*				
Low	75 (64%)	1	1	
Moderate	163 (70.8%)	1 (0.62-1.61)	0.97 (0.59-1.59)	0.91
High	146 (67.7%)	1.1 (0.66-1.61)	1.16 (0.68-1.98)	0.57
Thrombocytopenia (platelets < 150000/mm^3^) *n* = 386				
*Age group*				
<15 years	306 (50.6%)	1	1	
≥15 years	80 (68.9%)	2.16 (1.41–3.3)	2.32 (1.5-3.6)	<10^−3^
*Gender*				
Male	208 (42.45%)	1	1	
Female	228 (54.15%)	1.05 (0.78-1.42)	1.1 (0.81-1.5)	0.52
*Parasitaemia*				
Low	39 (33.33%)	1	1	
Moderate	116 (50.4%)	2.03 (1.28-3.2)	2.01 (1.26-3.2)	<10^−3^
High	231 (62%)	3.27 (2.1-5.07)	3.4 (2.18-5.3)	<10^−3^

**Table 4:** Hemoglobin and platelets changes from day 0 to day 7 by treatment arms.

Treatment group	Point estimate changes of biological parameters from day 0 to day 7 (95% CI)	*p* value	Mean difference (95% CI)	*p* value
*Hemoglobin (mean g/dl)*				
ASAQ	-0.062 ± 0.2 (-0.47–0.35)	0.76	ASAQ ≠ AL : 0.33	0.55
AL	0.27 ± 0.08 (0.1–0.44)	0.0018	ASAQ ≠ DHAPQ : −0.59	0.07
DHAPQ	-0.65 ± 0.14 (-0.94–-0.37)	<10^−3^	AL ≠ DHAPQ : −0.93	<10^−3^
ASMQ	0.54 ± (0.27–0.81)	<10^−3^	ASAQ ≠ ASMQ : −0.61	0.041
			AL ≠ ASMQ : −0.26	0.56
			DHAPQ ≠ ASMQ : −1.2	<10^−3^
*Platelets (count/mm^3^)*				
ASAQ	-155399 (-198437.3–-112361)	<10^−3^	ASAQ ≠ AL : 0.33	0.55
AL	-181761 (-198241–-165281)	<10^−3^	ASAQ ≠ DHAPQ : −0.59	0.07
DHAPQ	-217617 (-241079–-194154)	<10^−3^	AL ≠ DHAPQ : −0.93	<10^−3^
ASMQ	-185082 (-206793–-163370)	<10^−3^	ASAQ ≠ ASMQ : −0.61	0.041
			AL ≠ ASMQ : −0.26	0.56
			DHAPQ ≠ ASMQ : −1.2	<10^−3^

**Table 5:** Biochemical parameter changes from day 0 to day 7 by treatment arms.

Treatment group	Point estimate changes of biological parameters from day 0 to day 7 (95% CI)	*p* value	Mean difference (95% CI)	*p* value
*AST (IU/l)*				
ASAQ	12.8 ± 2.4 (5.4–7.3)	*<10^−3^*	ASMQ ≠ AL : −3.07	*<10^−3^*
AL	14.4 ± 1.6 (11.3–17.5)	*<10^−3^*	ASMQ ≠ DHAPQ : −7.2	*<10^−3^*
DHAPQ	7.15 ± 2.4 (2.4–12)	0.003	ASAQ ≠ AL : −2.1	*0.79*
ASMQ	17.6 ± 2.2 (13.3–22)	*<10^−3^*	ASAQ ≠ ASMQ : −3.07	*0.29*
			ASAQ ≠ DHAPQ : −4.1	*0.07*
			AL ≠ DHAPQ : −2.02	*0.6*
*ALT (IU/l)*				
ASAQ	9.3 ± 2.3 (4.8–13.8)	*<10^−3^*	ASMQ ≠ AL : 0.55	*1*
AL	7.6 ± 1.03 (5.6–9.6)	*<10^−3^*	ASMQ ≠ DHAPQ : 4.2	*0.02*
DHAPQ	3.15 ± 2.2 (−1.2–7.5)	*0.15*	ASAQ ≠ AL : 0.63	*1*
ASMQ	11.7 ± 1.5 (8.7–14.6)	*<10^−3^*	ASAQ ≠ ASMQ : −0.07	*1*
			ASAQ ≠ DHAPQ : 4.3	*0.04*
			AL ≠ DHAPQ : 3.6	*0.01*
*Creatinine (6-14* mg*/l)*				
ASAQ	3.5 ± 0.8 (1.7–5.2)	<10^−3^	ASMQ ≠ AL : −0.46	*1*
AL	0.16 ± 0.09 (−0.33–0.35)	0.1	ASMQ ≠ DHAPQ : −3.7	*<10^−3^*
DHAPQ	-0.08 (-0.3–0.13)	0.45	ASAQ ≠ AL : −3.4	*<10^−3^*
ASMQ	0.12 ± 0.1 (−0.12–3.7)	0.33	ASAQ ≠ ASMQ : 3	*<10^−3^*
			ASAQ ≠ DHAPQ : −6.7	*<10^−3^*
			AL ≠ DHAPQ : −3.3	*<10^−3^*
*Bilirubin (0.23-1* mg*/dl)*				
ASAQ	0.35 (0.17–0.52)	*0.0001*	ASMQ ≠ AL : −0.16	*<10^−3^*
AL	0.31 (0.2–0.43)	*<10^−3^*	ASMQ ≠ DHAPQ : −0.6	*<10^−3^*
DHAPQ	0.89 ± 0.14 (0.6–1.2)	*<10^−3^*	ASAQ ≠ AL : −0.46	*<10^−3^*
ASMQ	0.41 (0.29–0.54)	*<10^−3^*	ASAQ ≠ ASMQ : 0.3	*<10^−3^*
			ASAQ ≠ DHAPQ : −0.9	*<10^−3^*
			AL ≠ DHAPQ : −0.44	*<10^−3^*

## Data Availability

Data supporting the findings of this study may be released upon reasonable request to corresponding author.
